# Translating biomonitoring data into risk management and policy implementation options for a European Network on Human Biomonitoring

**DOI:** 10.1186/1476-069X-7-S1-S2

**Published:** 2008-06-05

**Authors:** R Smolders, G Koppen, G Schoeters

**Affiliations:** 1 VITO, Environmental Toxicology Department, Boeretang 200, 2500 Mol, Belgium

## Abstract

**Background:**

The "European Environment & Health Action Plan 2004–2010" originates from the concern of the European Commission on the well-being of individuals and the general population. Through this plan, the Commission has set the objectives to improve the information chain for a better understanding of the link between sources of pollution and health effects, to better identify existing knowledge gaps, and improve policy making and communication strategies. Human biomonitoring (HBM) has been included as one of the tools to achieve these objectives. As HBM directly measures the amount of a chemical substance in a person's body, taking into account often poorly understood processes such as bioaccumulation, excretion, metabolism and the integrative uptake variability through different exposure pathways, HBM data are much more relevant for risk assessment than extrapolations from chemical concentrations in soil, air, and water alone. However, HBM primarily is a stepping stone between environmental and health data, and the final aim should be an integrated and holistic systematic risk assessment paradigm where HBM serves as a pivotal point between environment and health, on the one hand leaning on environmental data to provide detailed information on the sources and pathways of pollutants that enter the human body, and on the other hand clarifying new and existing hypotheses on the relationship between environmental pollutants and the prevalence of diseases. With the large amount of data that is being gathered in the different national survey projects, and which is expected to become available in Europe in the near future through the expected European Pilot Project on HBM, a framework to optimize data interpretation from such survey projects may greatly enhance the usefulness of HBM data for risk managers and policy makers.

**Results:**

This paper outlines an hierarchic approach, based on the stepwise formulation of 4 subsequent steps, that will eventually lead to the formulation of a variety of policy relevant risk reduction options.

**Conclusion:**

Although the usefulness of this approach still needs to be tested, and potential fine-tuning of the procedure may be necessary, approaching the policy implications of HBM in an objective framework will prove to be essential.

## Introduction

The "European Environment & Health Action Plan 2004–2010" originates from the concern of the European Commission on the well-being of individuals and the general population. While obviously our highly industrialized European society provides many of its citizens with a very high quality of life, there are also costs to this modern society in terms of increased pollution, noise, climate change and other stressors that may affect our every day quality of life. Hence, it should be a constant goal of scientists, risk managers and policy makers to maximize the profits from our highly evolved society while minimizing its health-related costs. Together with improving general public health, there are also indirect yet large benefits in terms of long-term economic growth and sustainable development, since the indirect costs in productivity losses due to illness or premature death may be substantially larger than the cost for direct health care [1-3].

Hence, even while stringent environmental legislation, at a local, national and European level, aims at minimizing the negative effects of our modern society, there remains a need to be on the outlook for existing, new and emerging stressors that may hamper the general population's health. It is therefore essential to continuously monitor our day-to-day environment and identify potential threats as soon as possible, to evaluate the effect of mitigating actions to deal with identified threats and to gather up-to-date information on the state of the environment in general [3,4].

Rather than merely looking at pollution sources, the Environment and Health Action Plan 2004–2010 considered a new approach to environmental policy making by revising and improving the health impact and risk assessment strategies [5]. In this context, three main themes are identified that provide a roadmap to a cleaner, healthier and better environment for its citizens. These themes are [1,2]:

1. Improving the information chain to understand the links between sources of pollution and health effects;

2. Filling the knowledge gap by strengthening research and addressing the emerging issues on environment and health;

3. Reviewing policies and improving communication.

Within the context of this paper, especially the first theme is of importance, with the perceived "*need for the development of a coherent approach to human biomonitoring in Europe*" presented specifically included in this theme as Action 3. Also themes 2 and 3 have identified actions with a direct relevance to the European Project on human biomonitoring, particularly through integrating European environment and health research (Action 5) and reviewing policies and improving risk communication. Human biomonitoring (HBM) is defined as 'the measurement of chemicals or their metabolites in human tissues or specimens, such as blood or urine' [6]. Until recently, the traditional way of describing these relationships was by estimating the concentration of chemicals in different environmental compartments using empirical or modeling efforts, taking into account human exposure estimates to quantify the dose. However, due to both an increase in analytical capacity and a change in social awareness towards pollution exposure, there has been a rapid increase in the development and application of human biomonitoring (HBM) as a tool to evaluate exposure (i.e. the use of human tissue or fluid samples to estimate exposure). In many cases, HBM data have been proven to be a valuable supplement, or have even surpassed, estimates of exposure based on environmental measures [7-9]. HBM directly measures the amount of a chemical substance or its metabolite in a person's body, taking into account often poorly understood processes such as bioaccumulation, excretion, metabolism and the integrative uptake variability through different exposure pathways, rather than each individual exposure source. Hence, these data are much more relevant for risk assessment than extrapolations from chemical concentrations in soil, air, and water [9-11]. As was phrased by Stokstad [12], "pollution gets personal" when HBM data are being collected. Not only does this relate to a change of perception in the general public, it also integrates environmental exposure in a way that is more likely to be consistent with health effects, as the causative compounds have actually entered the body and are still detectable [10]. However, presenting HBM data without a proper context may lead people to the understandable yet often incorrect assumption that low levels of chemicals found in our tissues are harmful, simply by their presence itself [9,13].

Within Action 3 of the Environment and Health Action Plan 2004–2010, the need for integration of HBM data with environmental and health data is specifically foreseen:

*"Biomonitoring is not an automatic instrument, which can be considered in isolation, but has to be integrated with environmental monitoring, toxicological and eco-toxicological data and especially with considerations related to analytical epidemiology*. [2]"

This statement highlights that human biomonitoring (HBM) is not an island on itself, but should be considered a stepping stone between environmental and health data [11]. The final aim should be an integrated and holistic systematic risk assessment paradigm where HBM serves as a pivotal point between environment and health, on the one hand leaning on environmental data to provide detailed information on the sources and pathways of pollutants that enter the human body, and on the other hand clarifying new and existing hypotheses on the relationship between environmental pollutants and the prevalence of diseases or the occurrence and identification of disease clusters [3,10,14].

## HBM and the environment and health paradigm

Most examples on the use of HBM as a source of information in the environment and health paradigm come from small-scale, well documented research projects. In these research projects, HBM data is considered a complementary source of information and data are gathered to provide more insight in the link between environment and health, with a focus on hypothesis generation and testing. Generally, the choice of biomarkers, matrix and identification of the studied population are based on an *a priori *research question, and choices are such that HBM data offer an optimal source of information with regard to the research question. This type of research project usually cover only a limited geographical area, or a targeted sub-group (pregnant women, children, etc.), a relatively limited number of participants and often address the relationship between environmental levels of contaminants and the resulting human exposure. Well-known recent examples in literature include the use of HBM to evaluate the effect of fish eating or amalgam fillings on mercury contamination [15-17], the effect of lead as an anti-knocking agent in gasoline [18-20], or the effects of anti-smoking laws on Cotinine levels [21-23]. Other research projects address the links between the levels of pollutants in the body and adverse health effects. Examples are the relationship between methylmercury and neurobehavioral deficits [24,25], between lead and intellectual performance of children [26,27], or between cadmium and effects on the kidneys [28,29]. These research topics allow the development of health based guidance values based on levels of pollutants in the body.

On the other hand however, there are a number of large-scale survey programs that have been developed independent of any specific research questions, and are generally aimed at providing researchers and policy makers with a broad picture of the pollutant load among the general population. These survey projects often cover broad geographical areas, include a wide variety of biomarkers, and aim at providing periodical measurements of the prevalence of exposure to environmental agents, with a view to develop and evaluate policies that protect the health of the general population. Examples of this type of large-scale survey programs can be found in the German Environmental Survey (GerES), the USA's Center for Disease Control (CDC) program on HBM, or the Flemish Human Biomonitoring program. Often, these survey programs are associated with large-scale surveys on physical and mental health, such as the NHANES study in the USA [30] or KIGGS in Germany [31]. Table 1 gives some details on some of the most extensive HBM survey programs currently in place.

**Table 1:** Details on some HBM survey programs

	# Compounds	Time frame	Number of participants^a^
CDC [30]	116	1999–2000	7970
	148	2001–2002	8945
GerES [15, 74]	17	1985–1986	2731
	25	1990–1992	3966
	32	1998	4645
	61^b^	2003–2006	1244
FLEHS	6–9^c^	2002–2005	4300
ESBIO^ad ^[32]	4	2008–2010	6480

^a ^The number of constituents may vary per compound. Numbers are given for cadmium as a proxy, because Cd measurements are easy and cheap and generally are measured in all participating constituents

^b^The human biomonitoring program of the last GerES (GerES IV) included:

Lead, cadmium, mercury in blood

HCB, 3HCHs, DDE, PCB 138, 153, 180, 28, 52, 101 in blood

Nickel, mercury, arsenic, uranium, nicotine, cotinine, 6 metabolites of organophosphates, PCP and 9 other chlorophenols, 5 PAH metabolites, 5 pyrethroid metabolites in urine.

In addition:

11 phthalate matabolites in urine

Bisphenol A in urine

Three additional PAH (like tetrol)

All in all 61 substances for human biomonitoring.

More substances in the additional programs on indoor air (VOC), dust (flame retardants), drinking water and biological indoor contamination (personal communication Becker and Kolossa, 2008)

^c ^Depending on the age group considered

^d ^The ESBIO program is a proposed program according to Action 3 of the Environment and Health Action plan 2004–2010

The interpretation of HBM data in research projects is often straightforward, since data were gathered specifically to provide information on *a priori *research question, and the project was set up in such a way to maximize data interpretability and usefulness. However, the same is not necessarily true for HBM survey projects, since they generally provide a broad overview of pollutant exposure in the general population, without focusing on predefined research questions. However, within the vast amount of data that is being gathered in the different national survey projects, and which is expected to become available in Europe in the near future (see further), a framework to optimize data interpretation from such survey projects may greatly enhance the usefulness of HBM data for risk managers and policy makers. Evidently, survey and research projects should as much as possible be linked, where the research projects develop new biomarker tools, insights and knowledge on the complex interactions between HBM, environmental exposure and the resulting health effects, which can later be tested at a larger scale in survey projects.

## Towards a European network on human biomonitoring

As was already introduced earlier, the Environment and Health Action Plan 2004–2010 specifically addressed the use of HBM to gain better understanding on the link between environment and health issues, with the intention to develop a permanent harmonized European biomonitoring system [1,2,11]. Such a system will allow better understanding of environment and health linkages and long term health effects an will be used as a tool for the development of further environmental policy. This intention was later confirmed by the 2006 Environment and Health Information Review and Implementation Plan [11] and the opinion of the Scientific Committee on Health and Environmental Risks [3].

As a first step towards the establishment of an EU network on HBM, the European Commission launched the Expert Team to Support Biomonitoring in Europe (ESBIO) project (2004–2006), which consists of HBM experts from several EU Member States, and Croatia. The four main objectives of the ESBIO project were:

1. Development of a coordinated approach for biomonitoring

2. Elaboration how biomonitoring results can be integrated most efficiently with environmental monitoring and registered health data

3. Develop strategies to communicate biomonitoring results to stakeholders

4. Elaboration of scenarios for the use of biomonitoring results for policy making

ESBIO's work is directed towards the technical preparation of a European Pilot Project on Human Biomonitoring, and regularly reports to the Implementation Group on human biomonitoring, the European Commission and the Consultative Forum on Environment and Health. ESBIO's work is used as a basis by the Implementation Group on human biomonitoring to formulate recommendations to the Consultative Forum on the technical, structural and organizational requirements of such a European Pilot Project. Briefly, in the Implementation Group's Third Recommendation to the Consultative Forum on Environment and Health, it was suggested that a European HBM network should focus on a limited number of well-known, relatively easy to measure biomarkers. Methylmercury in scalp hair, lead in blood, cadmium in urine and cotinine in urine of both women of childbearing age (22–50 years old) and their children, age 6–11 [32]. For as many Member States as possible, 120 mother-child pairs per Member State will be asked to donate the relevant blood, urine and hair samples. In theory, this would thus mean having a study population of 3420 mother-child pairs, if all Member States choose to participate. At this point in time, a call for tender has indeed been issued by the European Commission within the Seventh Framework Program (ENV2007.1.2.2.1: European network on human biomonitoring), as yet no details are available as to the granting of the project [11,33]. It is within this context and framework of the ESBIO project, as a prelude for the Pilot Project, that the current document was developed.

In the following, we present a multi-step approach, which consists of four consecutive phases that need to be addressed in order to make better use of HBM survey data and to improve interpretation of spatial and temporal variation and potential health effects. The main advantage of including such a multi-step approach in the study protocol from the start of the project onwards, is that it offers a framework for the objective and impartial interpretation of HBM data, and allows the interpretation process and related decision making to be accessible to all participants and the general public from the start of the study.

## Step 1: Are there differences among biomarkers in time or space?

Mainly due to privacy and statistical reasons, HBM values are most often not represented as individual values. Generally, there is at least some kind of aggregation of data. This aggregation can take many forms, most commonly based on age groups, gender, ethnicity, or, if spatial differences in biomarker values are of main interest, on a geographical level. Because it is of major importance that data in the EU Pilot Project on HBM is available at a similar scale as environment and health data in Europe, data will preferably be aggregated at an appropriate geographical level (for example according to the Nomenclature of Territorial Units for Statistics (NUTS) classification), and it will be possible to calculate basic descriptive statistics for each geographical area [14]. Because differences in certain factors, such as age, gender or smoking behavior can have a significant effect on the outcome of HBM measurements, the appropriate corrections will have to be made in order to be able to adequately compare the different geographical areas.

Addressing this first step is mainly descriptive, and only offers answers on which subgroups are most likely to be exposed, and allows comparison of individual results to the average exposure of the entire studied population. Collecting individual biomarker values in this first step also allows examination of the dose distribution among the population and to identify the variability in biomarker values (low and high doses). The distribution of biomarker values is more informative than the average values since it allows the identification the proportion of high exposure groups in the population. However, this first step does not necessarily provide any information on risk or hazard of the biomarker studied, nor give information on possible health effects, suspected sources of exposure, or policy options to reduce exposure. The first step aims at aggregating and assembling data in a geographical framework, and offers constituents an idea of their pollutant load and the distribution of pollutants among the general population, compared to other regions, other people, or compared to previous monitoring programs in the same or different areas. Preferably, data will be presented in the form of maps, immediately highlighting regional differences. From a policy point of view, screening data can identify unusually high exposure which can serve as an early warning for unknown pollution sources or pollutants, even if the health impact of exposure at a given level cannot immediately be identified [11]. If HBM survey programs are repeated over time (see table 1), temporal evolutions can be derived. Not only does this provide scientists with information on possible new and emerging chemicals, but it can also provide information on the efficiency of policy measures taken to anticipate a potential threat [34]. For example, brominated flame retardants have been shown to increase dramatically in the breast milk of Swedish women over the last 30 years [35,36], which in itself was considered reason enough to ban specific polybrominated diphenyl ethers.

## Step 2: Are the observed differences reason for concern

It should be stressed that in the process of dealing with step 1, there is generally no information on the toxicity or potential hazard of substances included in the assessment, unless in cases where clear dose-effect relationships are known. In the example of brominated flame retardants, Darnerud et al [36] concluded that there was only very limited evidence of toxicity in humans was available, and also epidemiological studies in occupationally exposed workers found no consistent adverse effects attributable to exposure to these chemicals. However, the toxic potency of these chemicals was well demonstrated in animal experiments and the strong temporal increase in PBDE levels in the human population was considered a strong enough message to trigger risk manager and policy maker action.

Such strong and swift actions however are rare. Generally, temporal or spatial increases are less obvious, and more stringent rules need to be taken into account to assess the hazard of biomarkers detected in different matrices. Hence, after aggregation and appropriate correction of the data, the appropriate HBM values (average, median, x^th ^percentiles (P_x_) such as P_75_, P_90_,...) are compared with available benchmark or reference values in order to identify deviations. These values can be identified using a number of different approaches:

**Health-based legally binding standards**: Currently, there are no legally binding standards for internal exposure concentrations due to environmental exposure to substances. However, occupational exposure research has led to the development of e.g. "Threshold Limit Values" and "Biological Exposure Indices (BEI)", health-based guidance values for occupational exposure that can give an indication on the maximum tolerable intake of toxicants. BEI values for cadmium and lead were set at 5 μg Cd/L blood or 5 μg Cd/g creatinine in urine, and 30 μg Pb/dL in blood [37,38]. The European Union has set indicative occupational exposure limits (IOELVs), which for nicotine was set at 0.5 mg/m^3 ^for an 8 hour exposure period [39], and the binding biological limit value for lead and its organic compounds was set at 70 μg/100 mL blood, with medical surveillance needed at individual levels above 40 μg/100 mL blood, which was in 2002 lowered to 30 μg/dL, although it was mentioned that exposure to fertile women should be minimized [40]. However, these exposure limits are based on occupational exposure, and do not necessarily reflect adequate protection levels for environmental exposure;

**Threshold values**: A number of international agencies have developed guidelines, based on scientific research and expert judgment, for compounds where adequate consensus is available on the occurrence of health effects at certain concentrations. For example, WHO has developed health based guidance values for among others lead (10 μg/dL blood). Another good example of these values are the German HBM-I and HBM-II data, which have been established for a number of key pollutants (Table 2); The Human Biomonitoring Commission (HBC) of the German Federal Environment Agency recommends two different HBM values: HBM-I, the concentration of an environmental toxin in a human biological material below which there is no risk for adverse health effects, and HBM-II, the concentration above which there is an increased risk for adverse health effects in susceptible individuals of the general population and, consequently, an urgent need to reduce exposure and to provide individual biomedical advice;

**Table 2:** Examples of German HBM-I and HBM-II values for environmental pollutants

Matrix/Pollutant	Target population	HBM-I	HBM-II
Lead in blood	Females (18–45) and children < 12	100 μg/L	150 μg/L
	Females > 45 and males 18 – 69	150 μg/L	250 μg/L
Mercury in blood	Adults (18–69)	5 μg/L	15 μg/L
Cadmium in urine	Adults (18–25)	1 μg/g creatinine	3 μg/g creatinine
	Adults (26–69)	2 μg/g creatinine	5 μg/g creatinine

The Commission on Human Biological Monitoring of the German Federal Environmental Agency established in 1993 recommends two different HBM values: HBM I, the concentration of an environmental toxin in a human biological material below which there is no risk for adverse health effects, and HBM II, the concentration above which there is an increased risk for adverse health effects in susceptible individuals of the general population

**Data from international literature**: Based on internationally reported data, it is possible to compare European biomonitoring data with data collected in other parts of the world. For example, every two years the US-CDC publishes their 'National Report on Human Exposure to Environmental Chemicals", providing a wealth of information on the pollutant concentrations among different population groups in the US [30]. The current report provides HBM data on 148 chemicals, including Pb, Cd, Hg, and cotinine, for more than 6000 inhabitants. Reference ranges have been calculated from these substances, indicating the values into which 95% of the investigated population fall. They can be used to identify subjects with an increased level of exposure (in relation to background exposure) to a given environmental toxin but they do not represent health-related criteria for the evaluation of human biological monitoring data. The data merely provide toxicologists and physicians with a reference range so that they can determine whether or not people have been exposed to higher levels of a particular chemical than are found in the general population. These data will also help scientists to plan and conduct research about exposure routes and potential health effects [30]. Also, the German Commission on Human Biological Monitoring develops scientifically based criteria for the application and evaluation of HBM data. From the repeated HBM programs (see table 1), reference values, defined as the 95^th ^percentile of the distribution of concentrations of a specific compound in a matrix of a reference population, are calculated as a basis to identify individuals with an increased level of exposure [41,42];

**Historical data**: In several European Member States, there is already a wide variety of HBM data available from previous sampling programs, both research and survey programs. These data can serve as benchmark values, especially to detect changes in toxicant concentrations over time. For example, Germany has a history in HBM programs, with the first German Environmental Survey (GerES) reaching back as far as 1985–1986;

**Calculated reference values**: Based on the data obtained in the Pilot Study, reference values and ranges can be calculated for Europe as well as for the individual participating countries. HBM values from different countries or geographical areas can be compared to these calculated references. This will give an indication which areas have pollutant concentrations above the European average, and which are below this average. Apart from the average, also the P_90_-value is very useful to identify areas with high concentrations of a certain pollutant. The determination of reference values for the whole of Europe is one of the aims of the EU Pilot Project on HBM, which can be used as benchmark values to identify areas with unusually high exposure, or to detect future trends in pollutant [11].

There is generally no objective criterion to assess whether "observed differences are a reason for concern" (step 2). For a few chemicals, there are clear, internationally accepted health based reference values (table 2), but for the most part, there is no such benchmark available. As argued earlier, simply detecting the presence of a chemical in a biological matrix should not be confused with an increased risk [4]. Hence, the establishment of an Expert Panel is proposed as the most impartial way to identify, evaluate, and rank biomarker data with respect to their toxicity and concern for human health. This Expert Panel should be a multi-national group of scientists with experience or expertise in (a specific part of) the biomarker under evaluation (e.g toxicologists, epidemiologists, chemists, medical doctors). They should form an opinion on the scientific relevance of the biomarker levels under consideration, and should be able to differentiate, prioritize, and evaluate whether observed differences are a reason for concern.

## Step 3: Can potential (local) sources be identified?

Generally, both HBM research and survey projects gather considerably more information than simply sampling and analyzing biomarkers in a specified matrix. As already mentioned earlier, HBM survey projects are often part of a larger study on environment and health, and as in general, participants are requested to fill in a more or less extensive questionnaire. These questionnaires are constructed in such a way that they provide researchers with detailed information on the socio-economic background, food preferences, hobbies, occupational exposure patterns, and potential local sources of recent exposure. By careful analysis of the questionnaire data, researchers often are able to pinpoint exposure sources to non-environmental factors (e.g. lifestyle or work), local or individual environmental factors (e.g. indoor air quality, food preferences) or general environmental factors. Examples in literature on how behavior may alter biomarker values include the observation that a diet rich in fish and amalgam fillings are generally the major source of MeHg exposure [15,16], how consumption of free-range chicken eggs may often result in substantially higher dioxin and dioxin-like PCB doses than consumption of barn or cage eggs [43], or how smoking restrictions have an impact on urinary cotinine levels in non-smokers [44-46].

Moreover, to further refine the potential answers to this third step, additional information such as pollutant concentrations measured in animals (e.g. ecosurveillance), local environmental data from different environmental compartments gathered through a variety of regional or national monitoring networks with subsequent pollution dispersal modeling, or industry pollution licenses may prove highly valuable to provide more insight in local sources of potential exposure. Ecosurveillance data from the International Council for the Exploration of the Sea (ICES) or OSPAR may provide additional information on spatial patterns on mercury levels in seafood [47,48], or pollution emission inventory databases like EPER (European Pollution Emission Register, [49] may provide high quality and EU-wide information on potential sources of contaminant emission and exposure.

## Step 4: How to translate this information to policy makers and risk managers?

Generally, as an outcome of the process of going through the three previous steps, there are three options on how HBM data can be translated into advice for risk managers or policy makers. A first option is that the higher exposure in either time or space is due to (local) emission sources, in which case policy makers should take measures to develop and implement risk reduction strategies. Obviously, this cannot be done solely based on toxicological information alone, and a multi-disciplinary weight-of-evidence approach, in which toxicological, socio-economic, political and stakeholder arguments are balanced in order to optimize societal benefits (see further). One of the most obvious examples of this option is the Stockholm Convention on Persistent Organic Pollutants (POPs) (see  for more information). This groundbreaking agreement aims at restricting or eliminating POPs from production, and was heavily supported by biomonitoring data, in that many of these POPs were globally detected in blood, fat and breast milk [50-52]. Next to driving new policy regulations, HBM can also be used to evaluate the efficacity of existing regulations. Tobacco laws aim to restrict exposure of the population to tobacco smoke and hence reduce the health risks. Monitoring cotinin in the urine of children, which is a tracer of exposure to passive smoking, will allow to evaluate whether implementation of these laws in different countries has been successful in terms of reducing exposure to a vulnerable population.

HBM will be especially relevant as a follow up tool for the new ambitious REACH program regarding the implementation of a new chemicals policy in the EU, which aims at a better protection of the environment and human health in the EU. Human biomonitoring is a unique tool to evaluate the efficacy of the program, provided that validated biomarkers for the chemicals under consideration are available.

A second option is that higher exposure may be due to lifestyle factors, in which case the best way forward is to alter consumer behavior or lifestyle [53]. Though in this option, individual constituents may be the key to altering exposure patterns, a role is also foreseen for policy makers. Through informing and sensitizing the general audience, policy makers and risk managers can have an important impact on the exposure profiles. Recently, the Flemish Government started a campaign to collect previously banned pesticides such as DDT. While these compounds are banned in Flanders since 1974, the Flemish HBM program still detected elevated DDE-concentrations in rural areas in Flanders. With exposure assumed to be mainly due to individual behavior of constituents, the government used HBM data to inform and sensitize the general audience and to adapt environment and health policies.

Finally, a third option concludes that no definitive verdict can be made on the potential source of exposure, in which case more research will be necessary.

Very often, there are considerable data gaps in the presence of detailed, relevant, and up to data toxicological data. Paustenbach and Galbraith [9] argue that for most of the > 200 chemicals monitored by the CDC and other organizations, relating chemical exposure to measurable health risks is problematic, and more research is essential to properly inform the public. As an answer to this general lack of reliable toxicity data, the European Commission has recently approved the REACH legislation (Registration, Evaluation, and Authorisation of Chemicals) [34].

In this last case, when more research is needed to make a well-balanced proposal towards policy makers and risk managers, going through the four questions process will allow the identification of data gaps, which in itself already allows sound advice to be given towards future research and policy efforts.

Translating HBM information into risk management and policy making options (step 4) is not a straightforward procedure. While the Expert Panel described under step 2 can be a multinational consortium of toxicologists, translating HBM information into risk management requires a multi-disciplinary approach and is mostly region- or nation-specific. Therefore in order to provide expert judgment for step 4, a multidisciplinary Stakeholder Task Force is proposed. The Stakeholder Task Force needs to evaluate the evaluations and arguments of the Expert Panel, and also takes into account political and socio-economic arguments in a weight-of-evidence approach. It is essential that the Stakeholder Task Force can assimilate the mainly scientific advice of the Expert Panel, and combine it with other issues such as general well-being of the population, differences in susceptibility of sub-populations or specific windows of vulnerability, policy priorities, past actions, financial and political possibilities and public concern. The Stakeholder Task Force therefore should not merely consist of medical and toxicological scientists, but should also contain administrators, policy makers, social scientists, representatives of Non-Governmental Organizations etc. They will need to face the task of balancing different lines of evidence, weigh them, and integrate them all into one recommendation that is suitable for policy support and risk management.

## Risk reduction strategies

Eventually, the four-step procedure outlined above culminates into the formulation of one or more proposals to risk managers and policy makers to control the risk of the environmental stressors under consideration. A number of risk reduction strategies are possible, and are discussed in more detail below:

**No immediate action needed**: obviously, the outcome of the four-step procedure may be that based on the current knowledge on the link between environment, dose and health, taking into account the appropriate safety factors and the precautionary principles, no immediate threat of environmental pollutants for human health is expected. It remains important however to clearly document and communicate the scientific, socio-economic and political elements in the decision making process, because this might be useful for later repeated risk assessments or the evaluation of future additional or conflicting information;

**Awareness raising**: While a large portion of the general population often is aware of the threat of major environmental stressors such as heavy metals, PCBs or dioxins, there often is only limited awareness among the general public on potential threats of many ubiquitous substances that have attracted little or no media attention. Furthermore, media attention may be biased towards alarming messages on the presence of chemicals in the environment. Scientists, policy makers and stakeholders should develop clear messages on the presence of chemicals in the environment, disseminate objective, unbiased and detailed knowledge on potential exposure routes, health effects and risks and propose simple yet efficient guidelines for the general audience to control their own exposure and health risks. A number of studies have illustrated how HBM can be effective in creating better knowledge on exposure routes of contaminants, and hence the possibility to use this information to promote alternative lifestyles or to change individual behavior. For example, the negative effect of fish consumption on the methylmercury body burden and related health effects is well supported by HBM data, especially for pregnant women and small children [54-56]. However, fish consumption also has a number of positive effects because of its nutritional composition, such as the effects of omega-3 fatty acids on the quality of a pregnancy and the neurodevelopment of the infant [57,58]. By integrating all this knowledge, creating awareness in the form of advice on fish consumption patterns can maximize the nutritional input from fish while minimizing the detrimental effects of pollutants [55]. Based on this HBM-driven knowledge, several government agencies globally recommend for pregnant women, women of childbearing age, and children under 15 [59,60], among others:

◦ To mainly eat those fish species that have naturally low mercury levels;

◦ To limit or ban the consumption of fresh shark, swordfish, or tuna;

◦ To check local advisories on locally caught fish, e.g. by sports anglers

**Controlling substance exposure**: Raising awareness may be an efficient way to control exposure and hence risk of hazardous exposure, but remains a voluntary action by individual concerned citizens rather than a harmonized action by policy makers to protect broader groups of citizens. Mainly by using legislative tools, policy makers can better control the exposure of the broad public to potential health risks of environmental contaminants, if the need occurs. Rather than simply banning substances (see further), restricting the use of products under certain conditions may be an efficient controlling mechanism to limit the exposure of target populations or to limit general exposure. A textbook example to illustrate how "controlling substance exposure" can be a policy strategy is tobacco legislation in response to exposure to environmental (or second-hand) tobacco smoke (ETS). As has been illustrated many times, ETS can be biomonitored by measuring cotinin levels in urine, which serves as a biomarker for ETS-related health effects [61,62]. According to Wikipedia, the only country in the world to ban both the selling and smoking of tobacco products is Bhutan. For all other nations, smoking is not banned, but active smoking is mainly discouraged (placing it more in the "awareness raising" category of risk reduction options) and policy actions are undertaken to control exposure of non-smokers to second-hand smoke. In 2002, the European Council adopted the Council Recommendation on the prevention of smoking and on initiatives to improve tobacco control. In this recommendation, it is specifically mentioned that Member States should aim to protect smokers and non-smokers from ETS, given the health risks associated with passive smoking [39]. Many European Member States have accordingly developed an anti-smoking legislation, aimed at maximally reducing the exposure to ETS for non-smokers. Recent evaluations of national and statewide anti-smoking legislation, both in the workplace and other areas, shows that such legislation results in a significant reduction of exposure, as well as general improvements in respiratory and cardiac health [63,64];

**Banning substances**: Banning substances is without any doubt the most effective, yet also the most restrictive and invasive policy action available. Banning substances can be enforced by policy makers by ruling out the use of compounds, or may be voluntarily administered by industry through any form of pressure from policy makers or the general audience, promoting the use of alternatives, or simply by appealing to the good sense of any sector of daily life responsible for emission of the substance. On May 17^th ^2004, the Stockholm Convention on Persistent Organic Pollutants (POPs) entered into force. The Convention contains obligations on elimination and restriction of a number of chemicals that are a matter of concern due to their ubiquitous occurrence, persistence, bioaccumulation and potential health effects on nature and humans. While the list of chemicals identified as POPs was mainly developed based on ecological monitoring data instead of HBM data, HBM monitoring programs have proven extremely useful to evaluate the efficiency of the Stockholm convention and to monitor the decrease of POPs in the general population [65,66]. Also, HBM survey programs are aimed at identifying new and emerging POPs that may pose a threat on human health and for which legislative action may be undertaken, as is the case for among others brominated flame retardants [67,68]. Also industry can decide to phase-out chemicals which are suspected to pose a threat on environmental and public health, as was done for perfluorooctanyl sulfonate by 3 M in the year 2000 [69,70].

**Increased monitoring efforts**: while this strategy can overlap with "Further research" as a risk reduction strategy (see further), increased monitoring efforts does not aim at improving knowledge on the link between exposure and health effects, but merely aims at closely monitoring temporal or spatial trends of exposure or the development of health effects. Especially for biomarkers with a relatively short half-life, extreme biomarker values may be due to special, temporary circumstances and may not reflect the overall average exposure of constituents. Hence, increased monitoring efforts, including repeated sampling of persons with high exposure or extension of expanded sampling programs to include more participants (e.g. to better identify or validate hot-spot exposure) may be a relevant policy option. For example, in the case of chromium biomonitoring for occupational exposure, an expert panel suggested that a second or if necessary, a third spot (or 24-hour) urine sample was needed before it could be concluded that a person may be routinely overexposed [71]. For short-lived chemicals such as volatile organic compounds or agricultural pesticides, peak exposure may be due to infrequent exposure episodes and hence not representative for average exposure. Repeated sampling of high-exposure subjects provides more insight on the true nature of these high-exposure episodes [72]. Increased HBM efforts may also prove useful to better identify and delineate areas of high exposure, so-called hot-spots. Additional sampling may provide further information on the specific cause of exposure, and increasing the array of biomarkers measured may further clarify the link between exposure, dose, and response [73,74].

**Proxy monitoring**: while it is the intention of the steps outlined in the current document to convert HBM data into risk management and policy making options, the most efficient way of controlling human dose may not be by controlling human exposure, but by monitoring and controlling a specific, main, exposure route and setting limits for a proxy. This approach may be seen as a combination of awareness raising, controlling substance exposure and increased monitoring efforts, but generally involves ecosurveillance (or ecological biomonitoring) instead of human biomonitoring. For a number of well-studied chemicals such as cadmium or methylmercury, there are well-defined exposure sources that have a major contribution to the total dose. If more detailed information is needed on the spatial or temporal evolution of these compounds, an ecological biomonitoring network may be better suited to gather this information than a HBM network. Using animals or plants as a proxy for human exposure can alleviate some of the more troubling features of HBM, such as ethical issues, selecting representative subpopulations, correcting for confounding factors or statistical power requirements. For example, the use of lichens for monitoring of PCDD/Fs [75] or mosses for heavy metal monitoring [76] are documented in literature. HBM surveys have extensively documented that fish and seafood are a major source of methylmercury uptake in humans [16,17]. Hence, improved mercury monitoring may benefit from more detailed information on methylmercury content in seafood rather than providing additional data in human tissues, certainly if this type of data is directly fed into other policy options for risk reduction such as "controlling substance exposure" or "raising awareness" [77,78]. It needs to be stressed however that the use of proxy monitoring as a substitute for HBM is only valid if uptake routes are well understood. As already mentioned earlier, ecosurveillance data may in any circumstance be useful for the identification of potential (local) sources (step 3).

**Further research**: the absence of conclusive epidemiological evidence of the causal link between environmental stressors and health effects should not be a final argument to conclude that there is no health damage and hence preventive measures are unnecessary [53,79]. The limitations of epidemiological analysis and the uncertainty towards potential long-term, global health impairment have led to an increased acceptance and application of the precautionary principle. While environmental damage should be anticipated and prevented in the face of uncertainty by avoiding potentially damaging activities whenever possible, the precautionary principle should not be used as an endpoint of any assessment, but as a means to identify areas of high uncertainty, and thus areas in need of further research. In the past, the lack of high-quality toxicological data with clear dose-response relationships has frequently limited the efficiency of risk assessments, a situation that should improve drastically in the future with the REACH legislation coming into action [34]. However, also in other areas, further research efforts may substantially improve the relevance of risk assessments and reduce the level of uncertainty associated with them. Going through the higher identified four-step process will identify areas of high uncertainty, and will trigger potential new research topics.

## Conclusion

The establishment of a European Network on HBM will open new and exciting opportunities towards the collection of harmonized, comparable, high-quality data on the exposure and effects of environmental contaminants in humans. These data may provide relevant and useful information for risk managers and policy makers, in terms of developing, adapting and evaluating environmental policies. However, a framework is needed to put HBM data in perspective and to propose relevant policy adjustments.

This paper outlines a hierarchic approach, based on the stepwise formulation of 4 subsequent steps that will eventually lead to the formulation of a variety of policy relevant risk reduction options. Although the usefulness of this approach still needs to be tested, and potential fine-tuning of the procedure may be necessary, approaching the policy implications of HBM in an objective framework will prove to be essential.

## Competing interests

The authors declare that they have no competing interests.

## Authors' contributions

GK initially developed the Multi-step approach for application within the Flemish Biomonitoring Program. This approach was adapted by RS and GS to better suit application at a European level, and included the discussion on risk reduction strategies.
